# Supplementary material for: Getting blood from a stone: Improving brain–behavior inferences without brain data
Source: Imaging Neurosci (Camb). 2026 Sep 23;4:IMAG.a.1361. doi: 10.1162/IMAG.a.1361 (PMC13602129; doi:10.1162/IMAG.a.1361)
Supplement: Supplementary file 1 [file IMAG.a.1361_supp.pdf]

Supplemental Material for Getting Blood From a Stone: Improving Brain-Behavior Inferences Without Brain Data

David Halpern,[1*] Todd Gureckis,[1]

[1]Department of Psychology, New York University,

6 Washington Place, New York, NY 10003, USA

*Correspondence: david.halpern@nyu.edu

1 Application to Human Connectome Data

In order to test the performance of the Anderson $\hat{\rho}$ estimator, we conducted an analysis of brain-behavior correlations in the Human Connectome project (Van Essen et al., 2013) public data. Specifically, we investigated the relationship between total grey matter (FS_Total_GM_Vol) and performance on several cognitive tasks: The Variable Short Penn Line Orientation (VSPLOT_TC) (Benton et al., 1975; Gur, 2001), a two-back working memory task (WM_Task_2bk_Acc) (Cohen et al., 1994; Kirchner, 1958), the Penn Progressive Matrix Task (Gur, 2001; RAVEN, 1941), a language comprehension task (Language_Task_Acc) (Binder et al., 2011) and the age-adjusted composite Cognitive Function score from the NIH Cognitive Toolbox (CogTotalComp_AgeAdj) (Heaton et al., 2014). In order to generalize to a wider variety of task types and measures, we also included response times in a social-cognitive emotion task (Hariri et al., 2002) and grip strength (Reuben et al., 2013). In the full dataset of 1076 subjects with data on all variables, these correlations ranged in absolute value from .2 to .5. We created 50,000 bootstrapped datasets and analyzed the variance of estimated correlations using Pearson's r and the Anderson ρ estimator, assuming that the correlation on the full dataset was equal to the "true" population value (Fig. S1). As expected from the simulation analysis in Fig. 5, the Anderson estimator showed mixed performance, especially at low sample sizes. However at higher sample sizes, the Anderson estimator was able to improve over collecting additional neural data using just behavioral data. We note that these results do not perfectly match the simulations in the main text, likely for two reasons. First, the correlation estimate we present in parentheses is still estimated from the data and thus is measured with error. We therefore cannot perfectly align the simulations with the corresponding true correlation.

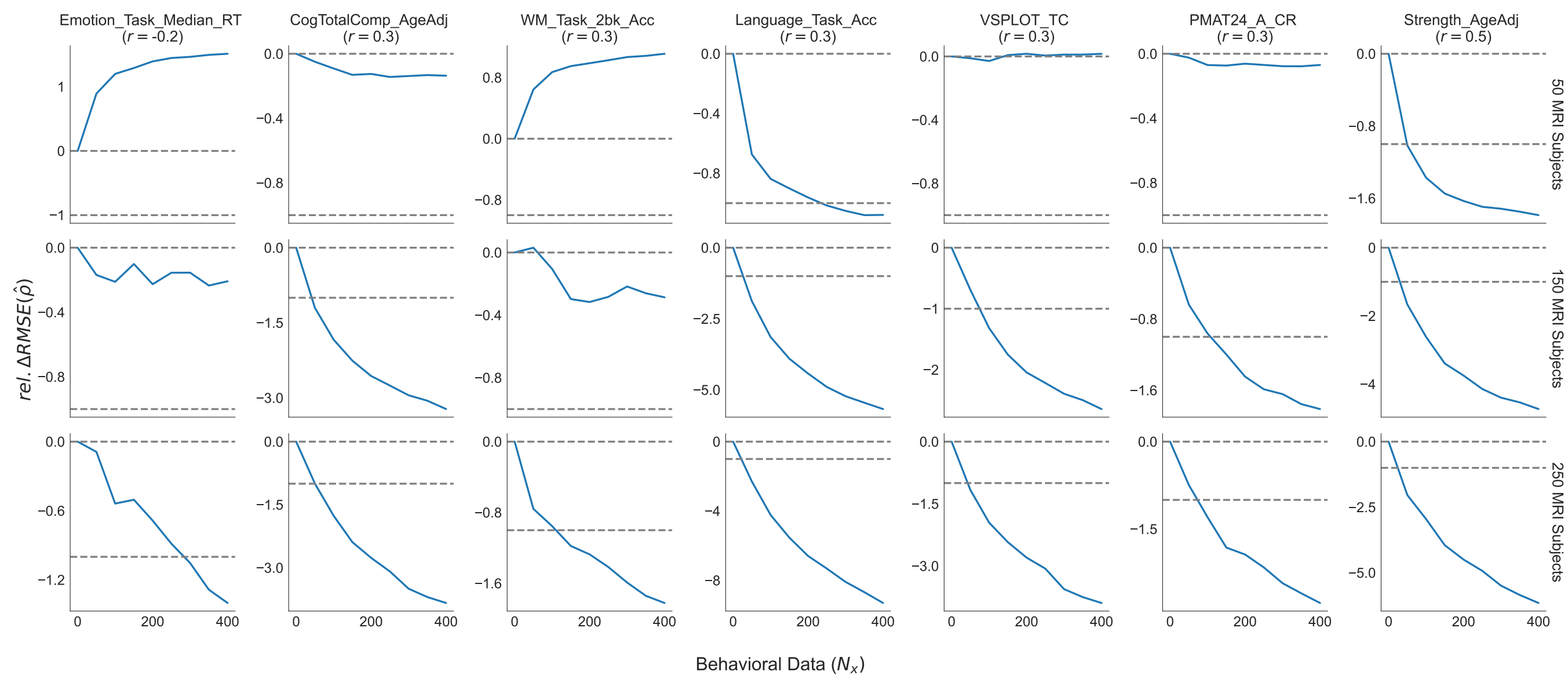

Figure S1: Relative decrease in root mean squared estimation error, averaged over 50,000 bootstrapped datasets, for the estimate of ρ as a function of the variable (with the correlation in the full dataset indicated in parentheses), the number of neuroimaging subjects N_{xy} and number of additional behavioral subjects N_x. Values are computed relative to the change in RMSE for a Pearson correlation with N_{xy} neuroimaging participants (0) compared to $N_{xy} + 1$ neuroimaging participants (-1). These values are indicated by the grey dashed lines. The solid line shows the Anderson (1957) $\hat{\rho}$ changes in the estimate as the number of additional behavior only subject are added to the analysis. Note that the axes here are not fixed across plots. This is necessary in order to see the change with number of subjects.

Second, while we can compute a correlation for arbitrary bivariate data, the actual data may deviate from the bivariate normal used in the simulations. For instance, the distributions in the HCP dataset may be highly skewed. Therefore, we would not expect the simulations to perfectly match these results. We hope that future work will sort out how to improve estimation at reasonable sample sizes.

2 Unbiased Anderson $\hat{\rho}$

As noted in the main text, the Anderson $\hat{\rho}$ estimator utilizes estimates of the variance of the behavioral variable with the N_{xy} subjects and the full $N_x + N_{xy}$ dataset. The maximum likelihood estimate, normalized by N is known to be biased and the unbiased estimate is normalized by $N - 1$. While all previous treatments of Anderson's ρ have used the biased MLE, we analyze the performance of the unbiased estimate in the simulation setup used for Fig. 5. We find that the unbiased estimate performs favorably, with decreased variance in the low N, low population correlation regime at the expense of slightly higher variance in the high N, high population correlation regime. Given that most cognitive

neuroscience studies are likely to be in the former, we recommend using the unbiased variance estimates and indeed that is what we do in all other simulations in the present work.

3 Absolute Change in Precision for Anderson's $\hat{\rho}$

While in the main text, we emphasize the benefit of behavioral data *relative* to an additional neuroimaging subject, it is also of interest to know the absolute change in precision when using the Anderson estimator. Therefore, Fig. S3 re-plots Fig. 5 in terms of root mean squared error.

References

Anderson, T. W. (1957). Maximum Likelihood Estimates for a Multivariate Normal Distribution when Some Observations are Missing. *Journal of the American Statistical Association*, *52*(278), 200–203. https://doi.org/10.1080/01621459.1957.10501379

Benton, A., Hannay, H. J., & Varney, N. R. (1975). Visual perception of line direction in patients with unilateral brain disease. *Neurology*, *25*(10), 907–907. https://doi.org/10.1212/WNL.25.10.907

Binder, J. R., Gross, W. L., Allendorfer, J. B., Bonilha, L., Chapin, J., Edwards, J. C., Grabowski, T. J., Langfitt, J. T., Loring, D. W., Lowe, M. J., Koenig, K., Morgan, P. S., Ojemann, J. G., Rorden, C., Szaflarski, J. P., Tivarus, M. E., & Weaver, K. E. (2011). Mapping anterior temporal lobe language areas with fMRI: A multicenter normative study. *NeuroImage*, *54*(2), 1465–1475. https://doi.org/10.1016/j.neuroimage.2010.09.048

Cohen, J. D., Forman, S. D., Braver, T. S., Casey, B. J., Servan-Schreiber, D., & Noll, D. C. (1994). Activation of the prefrontal cortex in a nonspatial working memory task with functional MRI. *Human Brain Mapping*, *1*(4), 293–304. https://doi.org/10.1002/hbm.460010407

Gur, R. (2001). Computerized Neurocognitive Scanning: I. Methodology and Validation in Healthy People. *Neuropsychopharmacology*, *25*(5), 766–776. https://doi.org/10.1016/S0893-133X(01)00278-0

Hariri, A. R., Tessitore, A., Mattay, V. S., Fera, F., & Weinberger, D. R. (2002). The Amygdala Response to Emotional Stimuli: A Comparison of Faces and Scenes. *NeuroImage*, *17*(1), 317–323. https://doi.org/10.1006/nimg.2002.1179

Heaton, R. K., Akshoomoff, N., Tulsky, D., Mungas, D., Weintraub, S., Dikmen, S., Beaumont, J., Casaletto, K. B., Conway, K., Slotkin, J., & Gershon, R. (2014). Reliability and Validity of Composite Scores from the NIH Toolbox Cognition Battery in Adults. *Journal of the International Neuropsychological Society*, *20*(6), 588–598. https://doi.org/10.1017/S1355617714000241

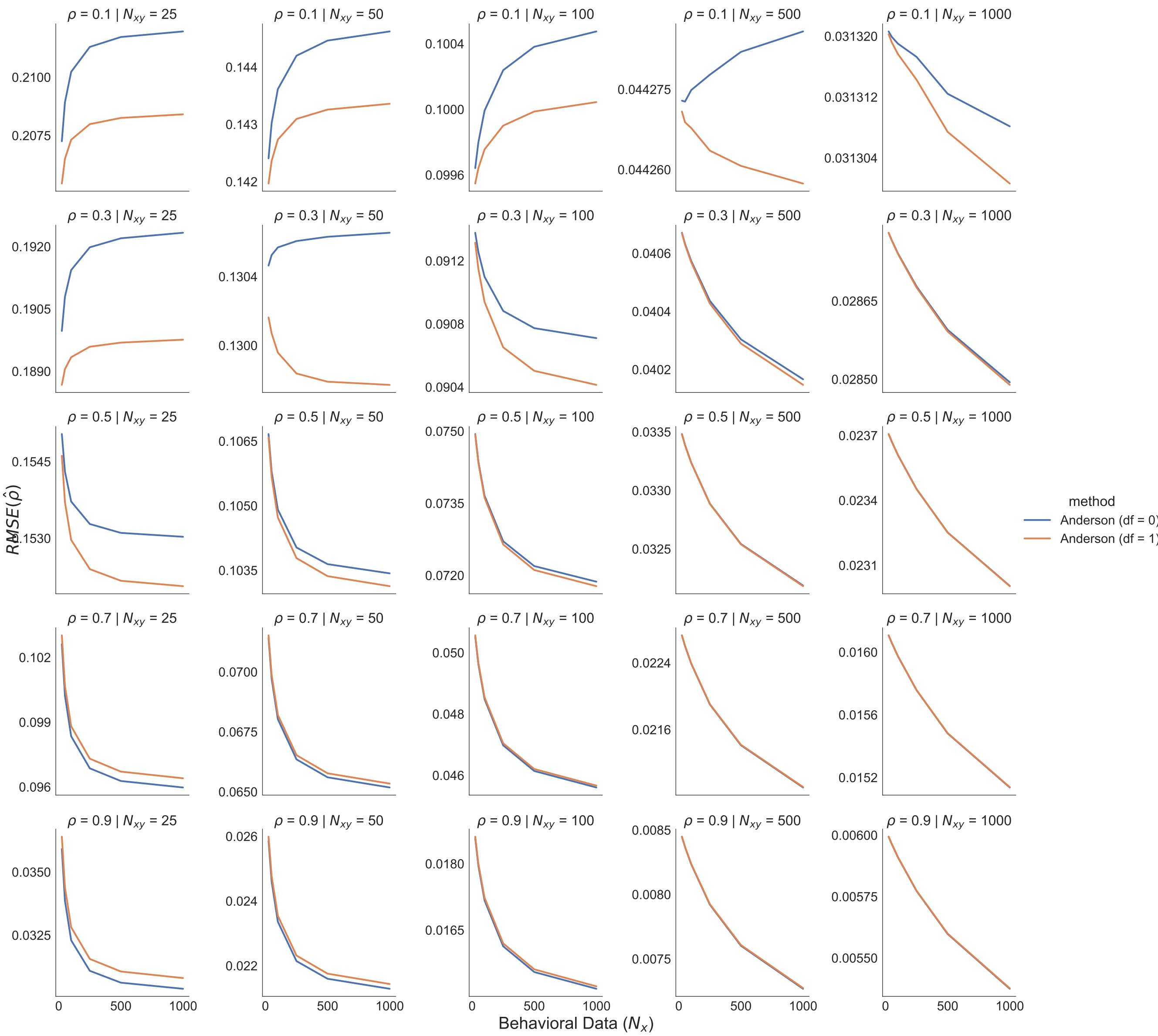

Figure S2: Root mean squared estimation error, averaged over 1,000,000 simulated datasets, for the estimate of ρ using the unbiased (df $= 1$) and biased (df $= 0$) estimates of variance, as a function of the true correlation, the number of neuroimaging subjects N_{xy} and number of additional behavioral subjects N_x. Note that the axes here are not fixed across plots. This is necessary in order to see the change with number of subjects.

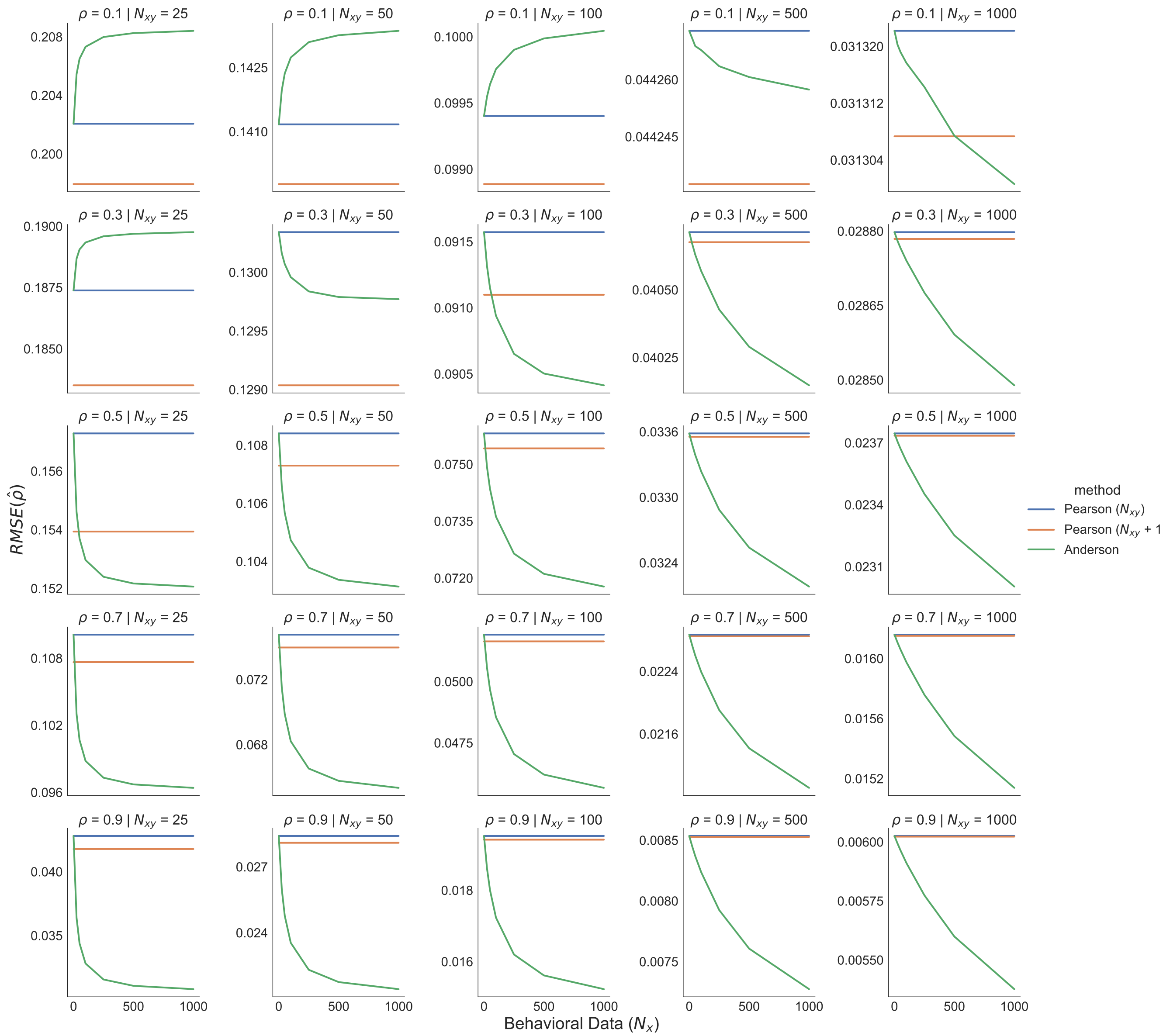

Figure S3: Root mean squared estimation error, averaged over 1,000,000 simulated datasets, for the estimate of ρ from Anderson (1957) as a function of the true correlation, the number of neuroimaging subjects N_{xy} and number of additional behavioral subjects N_x. As in Fig. 4, we also plot Pearson correlation with N_{xy} neuroimaging participants and $N_{xy}+1$ neuroimaging participants, indicated by the blue and orange lines. The green line shows the Anderson (1957) $\hat{\rho}$ changes in the estimate as the number of addition behavior only subject are added to the analysis. Note that the scale of the axes here are not fixed across plots. This is necessary in order to see the change with number of subjects.

Kirchner, W. K. (1958). Age differences in short-term retention of rapidly changing information. *Journal of Experimental Psychology*, *55*(4), 352–358. https://doi.org/10.1037/h0043688

RAVEN, J. C. (1941). STANDARDIZATION OF PROGRESSIVE MATRICES, 1938. *British Journal of Medical Psychology*, *19*(1), 137–150. https://doi.org/10.1111/j.2044-8341.1941.tb00316.x

Reuben, D. B., Magasi, S., McCreath, H. E., Bohannon, R. W., Wang, Y.-C., Bubela, D. J., Rymer, W. Z., Beaumont, J., Rine, R. M., Lai, J.-S., & Gershon, R. C. (2013). Motor assessment using the NIH Toolbox. *Neurology*, *80*(11_supplement_3). https://doi.org/10.1212/WNL.0b013e3182872e01

Van Essen, D. C., Smith, S. M., Barch, D. M., Behrens, T. E., Yacoub, E., & Ugurbil, K. (2013). The WU-Minn Human Connectome Project: An overview. *NeuroImage*, *80*, 62–79. https://doi.org/10.1016/j.neuroimage.2013.05.041
